# Meta‐analysis of echocardiographic quantification of left ventricular filling pressure

**DOI:** 10.1002/ehf2.13119

**Published:** 2020-11-23

**Authors:** Rachel Jones, Frances Varian, Samer Alabed, Paul Morris, Alexander Rothman, Andrew J. Swift, Nigel Lewis, Andreas Kyriacou, James M. Wild, Abdallah Al‐Mohammad, Liang Zhong, Amardeep Dastidar, Robert F. Storey, Peter P. Swoboda, Jeroen J. Bax, Pankaj Garg

**Affiliations:** ^1^ Department of Infection, Immunity and Cardiovascular Disease The University of Sheffield Sheffield S10 2RX UK; ^2^ Department of Cardiology Sheffield Teaching Hospitals NHS Foundation Trust Sheffield UK; ^3^ INSIGNEO, Institute for In Silico Medicine University of Sheffield Sheffield UK; ^4^ National Heart Research Institute Singapore National Heart Centre Singapore Singapore; ^5^ Bristol Heart Institute Bristol UK; ^6^ Leeds Institute of Cardiovascular and Metabolic Medicine University of Leeds Leeds UK; ^7^ Cardiology Directorate Leiden University Medical Centre Leiden The Netherlands

**Keywords:** Left ventricular end‐diastolic pressure, Echocardiography, Invasive heart catheterization

## Abstract

**Aims:**

The clinical reliability of echocardiographic surrogate markers of left ventricular filling pressures (LVFPs) across different cardiovascular pathologies remains unanswered. The main objective was to evaluate the evidence of how effectively different echocardiographic indices estimate true LVFP.

**Methods and results:**

Design: this is a systematic review and meta‐analysis. Data source: Scopus, PubMed and Embase. Eligibility criteria for selecting studies were those that used echocardiography to predict or estimate pulmonary capillary wedge pressure or left ventricular end‐diastolic pressures. Twenty‐seven studies met criteria. Only eight studies (30%) reported both correlation coefficient and bias between non‐invasive and invasively measured LVFPs. The majority of studies (74%) recorded invasive pulmonary capillary wedge pressure as a surrogate for left ventricular end‐diastolic pressures. The pooled correlation coefficient overall was *r* = 0.69 [95% confidence interval (CI) 0.63–0.75, *P* < 0.01]. Evaluation by cohort demonstrated varying association: heart failure with preserved ejection fraction (11 studies, *n* = 575, *r* = 0.59, 95% CI 0.53–0.64) and heart failure with reduced ejection fraction (8 studies, *n* = 381, *r* = 0.67, 95% CI 0.61–0.72).

**Conclusions:**

Echocardiographic indices show moderate pooled association to invasively measured LVFP; however, this varies widely with disease state. In heart failure with preserved ejection fraction, no single echocardiography‐based metric offers a reliable estimate. In heart failure with reduced ejection fraction, mitral inflow‐derived indices (E/e′, E/A, E/Vp, and EDcT) have reasonable clinical applicability. While an integrated approach of several echocardiographic metrics provides the most promise for estimating LVFP reliably, such strategies need further validation in larger, patient‐specific studies.

## Introduction

The primary pathophysiological process in heart failure (HF) is raised intracardiac pressure.^1^ For most patients with HF, this is associated with increased left ventricular filling pressure (LVFP) or left ventricular end‐diastolic pressure (LVEDP), caused by either systolic or diastolic impairment of the left ventricle (LV).^2^ LVEDP, a strong predictor of morbidity and mortality, has long been regarded as the key measurement for raised LVFP.^2^, ^3^, ^4^ Early diagnosis of raised LVFP is critical to inform the diagnosis of HF and also guide treatment optimization.^5^

**Figure 1:**
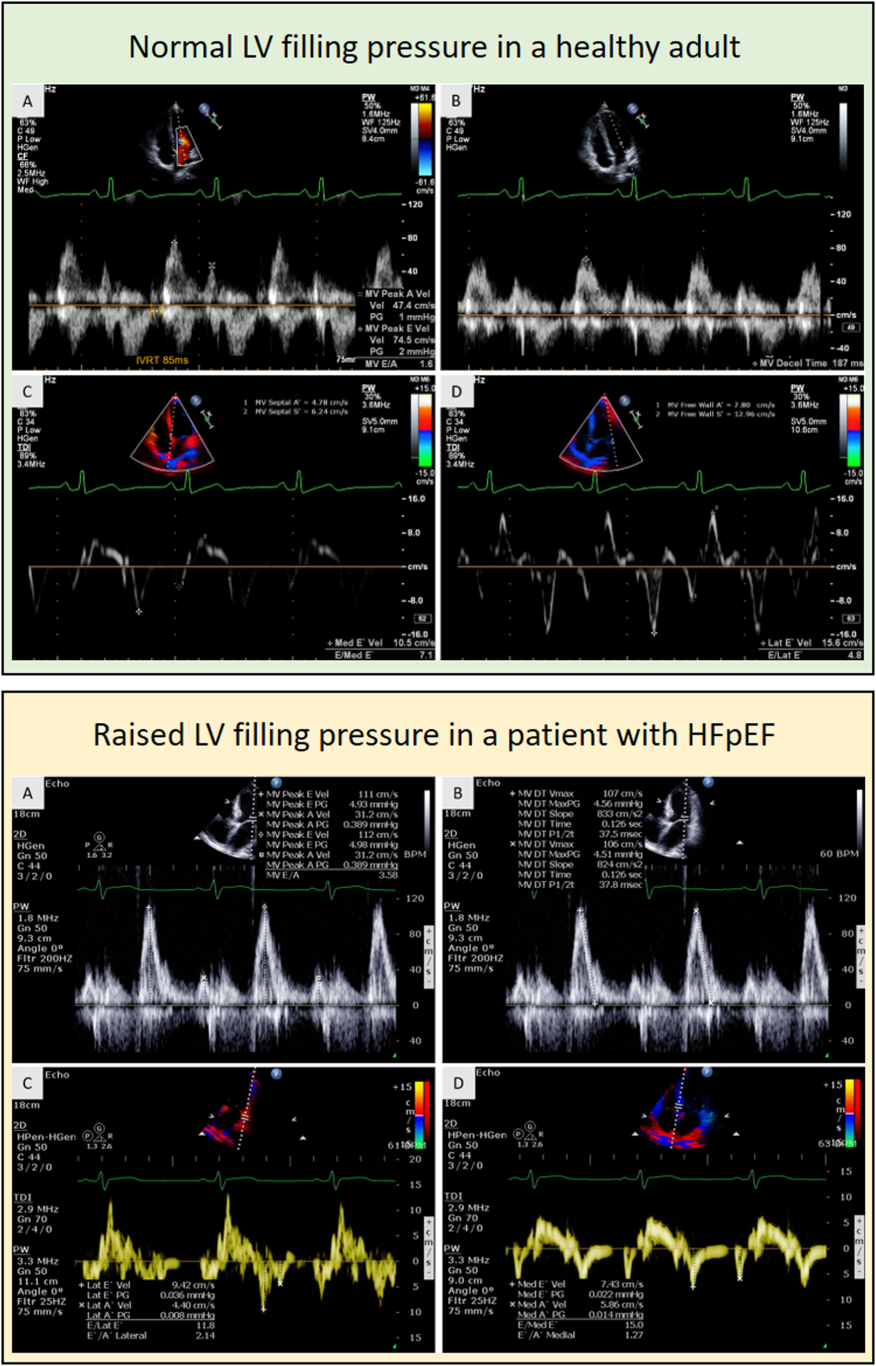
Case examples of Doppler‐based methods for left ventricular filling pressure (LVFP) assessment. The first case (green panel) is a healthy 20‐year‐old male; the second case (yellow panel) is a 60‐year‐old male patient admitted with heart failure (HF). In the healthy adult case, the E/e′ is <8 and suggests normal LVFP vs. in the patient, it is >12 and suggests raised LVFP.

The current standard approach to LVFP or LVEDP estimation is by transthoracic echocardiography (TTE). TTE allows for a detailed assessment of both LV diastolic and systolic function (*Figure* *1*).

Transthoracic echocardiography methods including mitral inflow, tissue Doppler annular velocities, tricuspid regurgitation velocity (TRV), and left atrial volume (LAV) have been widely studied to estimate LVFP. In particular, E/e′ has been the main focus of the majority of these studies. The current recommendations advocate sub‐categorization of patients depending on LV ejection fraction (LVEF). For patients with reduced LVEF (<50%), they recommend a focus on E/A ratio to estimate LVFP. And for those with preserved LVEF (>50%), a combination of E/e′, e′ velocity, TRV, and left atrial volume indexed for body surface area (LAVi) is advised to predict LVFP.^6^

The combined diagnostic accuracy of these TTE methods, to predict LVEDP in different HF states, including heart failure with preserved ejection fraction (HFpEF), heart failure with reduced ejection fraction (HFrEF), and even in valvular heart disease remains unclear. Systematic evidence synthesis is required to develop further understanding of which TTE methods are clinically applicable to predict LVFP in a specific HF state.

The aim of this systematic review and meta‐analysis was, therefore, to summarize current literature, systematically consolidate studies that have estimated LVEDP using TTE, and ascertain their predictive association with invasive measurements in different HF states.

## Methods

### Systematic review and meta‐analysis registration

This project was registered (CRD42020164642) with, and the ethics approved by, the prospective register of systematic review (PROSPERO).

### Eligibility criteria

Eligible studies were those that used TTE to predict or estimate pulmonary capillary wedge pressure (PCWP) or LVEDP. The primary endpoint was the agreement between invasive and non‐invasive methods. This was evaluated by both the correlation coefficient and statistical bias in the respective studies. This study mainly focused on the correlation coefficient (*r*), and all subsequent meta‐analyses were planned using *r* values. We limited our search to peer‐reviewed journals, medicine, and human adult (age ≥18 years) participants. Studies with fewer than 12 patients were excluded.

### Search strategy

The Scopus database, which incorporates all MEDLINE and Embase results, formed the basis of the literature search, undertaken on 21 November 2019. Search terms included ‘invasive’, ‘noninvasive/echocardiography’, and ‘LVEDP/Pulmonary Capillary Wedge Pressure/PCWP’. The full search strategy is included in the supporting information. An identical search of the Cochrane Central database returned no additional results. An extensive review of the referenced literature identified 11 additional studies. Duplicate results (*n* = 1) were removed, and the remaining results were combined (*n* = 207).

### Study selection

**Figure 2:**
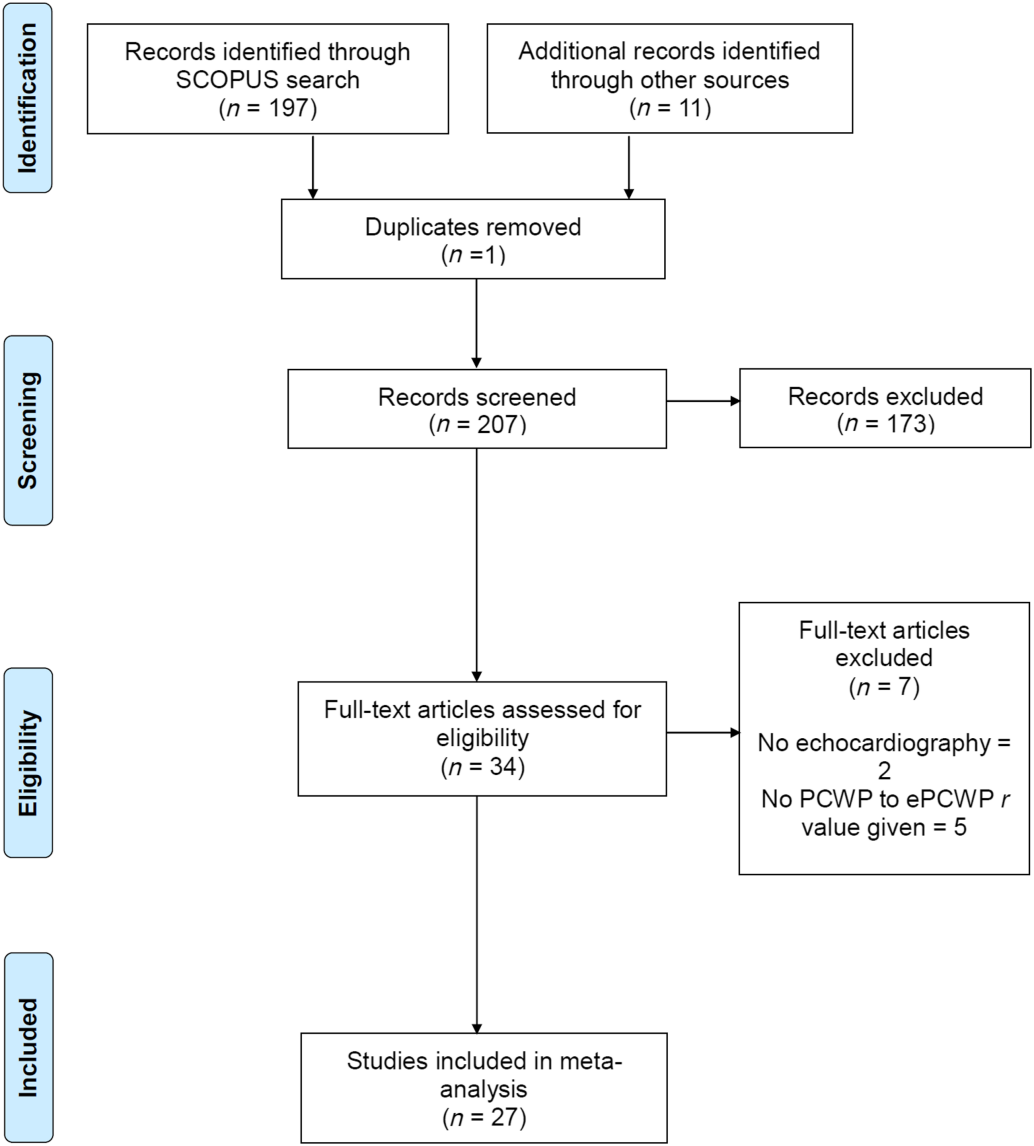
Search strategy flow chart as per the Preferred Reporting Items for Systematic Reviews and Meta‐Analyses 2009 guidance.

Preliminary vetting of the results, according to the Preferred Reporting Items for Systematic Reviews and Meta‐Analyses (PRISMA) guidelines, was undertaken by R. J. The shortlist was subsequently reviewed by an independent assessor, P. G. A third independent expert, A. J. S., was involved to resolve disagreements. The PRISMA flow chart is detailed in *Figure* *2*.

### Assessment of clinical applicability

We adapted the Critical Appraisal Skills Programme (CASP) tool to ensure clinical applicability to the evidence synthesis. This is detailed in the Supporting Information, *Tables* *S1* and *S2*.

### Statistical analysis and meta‐analysis

Statistical analysis was performed using MedCalc (MedCalc Software, Belgium, Version 19.1.5). We pooled the values of correlation coefficients and numbers recruited in each study for agreement between invasive and non‐invasive LVFPs. Detailed statistical methods are described in the supporting information.

## Results

The initial search identified 197 studies. A further 11 studies were identified through a thorough review of the referenced literature. After an initial screening of title and abstract, 34 research outcome papers were evaluated, and from these, 27 articles met the inclusion criteria^7^, ^8^, ^9^, ^10^, ^11^, ^12^, ^13^, ^14^, ^15^, ^16^, ^17^, ^18^, ^19^, ^20^, ^21^, ^22^, ^23^, ^24^, ^25^, ^26^, ^27^, ^28^, ^29^, ^30^, ^31^, ^32^ (*Figure* *2*). The total number of patients studied across all the 27 studies was 2058.

### Study characteristics

All studies were diagnostic cohort studies, using the invasive haemodynamic study as the reference test for LVFP. Consecutive patient recruitment was reported in 17 studies. Except for Nagueh *et al*.^27^ and Andersen*et al*.,^32^ all studies were single tertiary centre studies. All studies were published between 1997 and 2019. While some studies focused on specific disease states such as HFrEF (*n* = 8) and HFpEF (*n* = 11), some recruited heterogeneous patient populations (*n* = 11). From the 27 studies, only 8 studies (30%) reported both correlation coefficient and bias (Supporting Information, *Table* *S3*). The majority of studies (74%, 20/27) recorded invasive PCWP as a surrogate for LVEDP. Seven studies recorded LVEDP directly.^10^, ^14^, ^18^, ^22^, ^24^, ^30^, ^31^

### Meta‐analysis results for all studies

**Figure 3:**
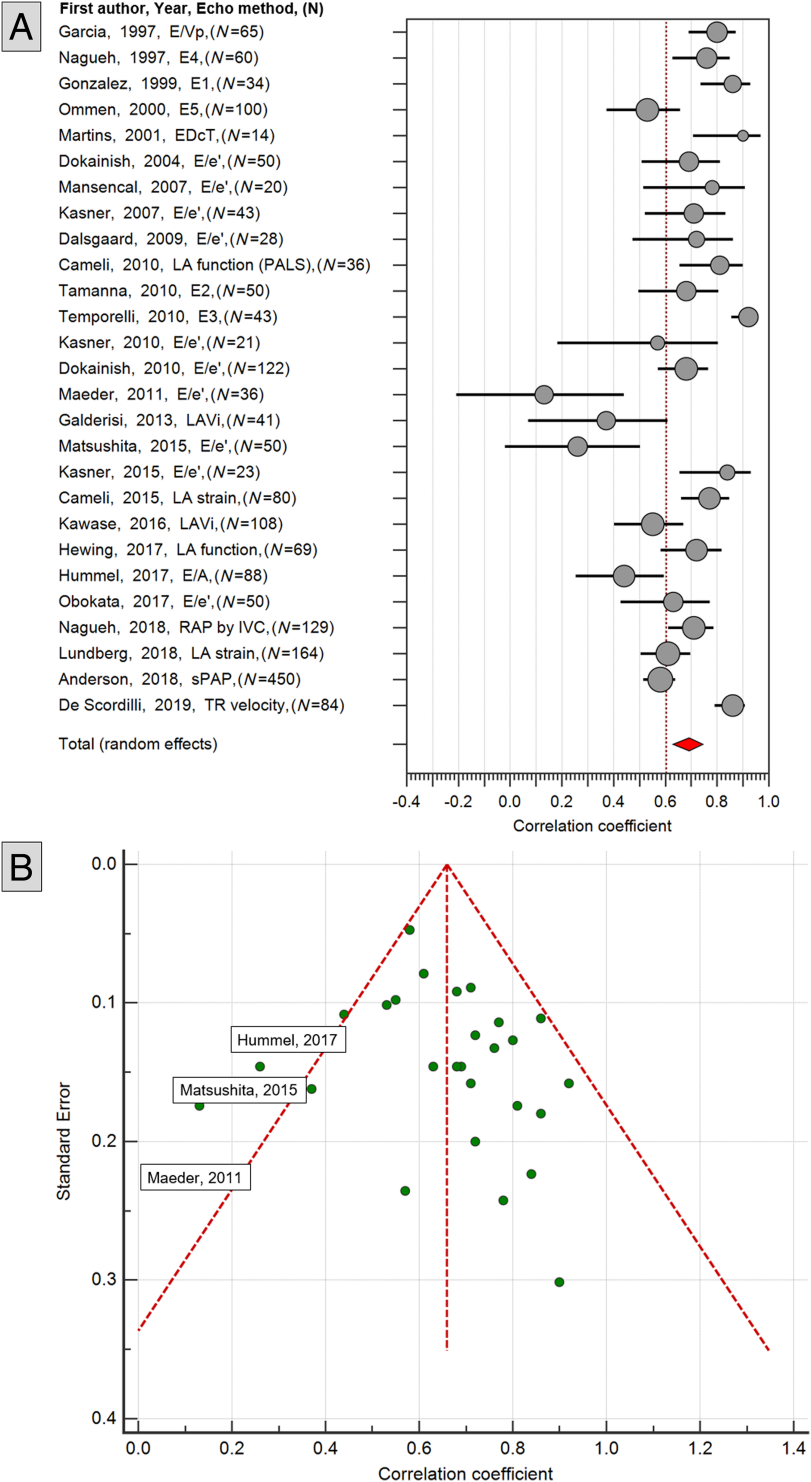
(A) Forest plot of all the 23 studies evaluated in meta‐analysis for non‐invasive assessment of left ventricular filling pressure. (B) Funnel plot of all the 23 studies.

The pooled analysis across all studies showed a statistically significant association of invasive haemodynamic assessment to echocardiographic methods—the weighted average (random effects) correlation coefficient for all the 27 studies was 0.69 [95% confidence interval (CI) 0.63–0.75, *P* < 0.01] (Supporting Information, *Table* *S4*). *Figure* *3* shows the forest plot of the pooled correlation coefficients, CIs, and percentage weighting for all studies included. Visual scrutiny of the forest plots suggests that a degree of between‐study variation exists in terms of the effect sizes. *I* ^2^ values of 81.6% confirmed significant levels of heterogeneity.

Temporelli *et al*. demonstrated the highest correlation coefficient with invasive measurement (*r* = 0.92, 95% CI 0.86–0.96), using an integrated mitral inflow velocity (MIV) equation (*Figure* *3*).^29^ However, they recruited only 43 patients, reducing the overall weight of the study. The study by Anderson *et al*. had the highest weight, closely followed by the Lundberg *et al*. work into left atrial (LA) strain.^32^, ^33^

Eight studies reported bias in the agreement between echocardiographic modelled LVFP vs. invasively measured LVFP. The maximum bias (bias = 9 mmHg) was reported by Maeder *et al*. in an HFpEF cohort (Supporting Information, *Table* *S3*).^19^ In HFrEF, the bias was comparatively less for the two studies by Cameli *et al*. and Temporelli *et al*.^16^, ^29^

### Meta‐analysis results for echocardiography metric

**Figure 4:**
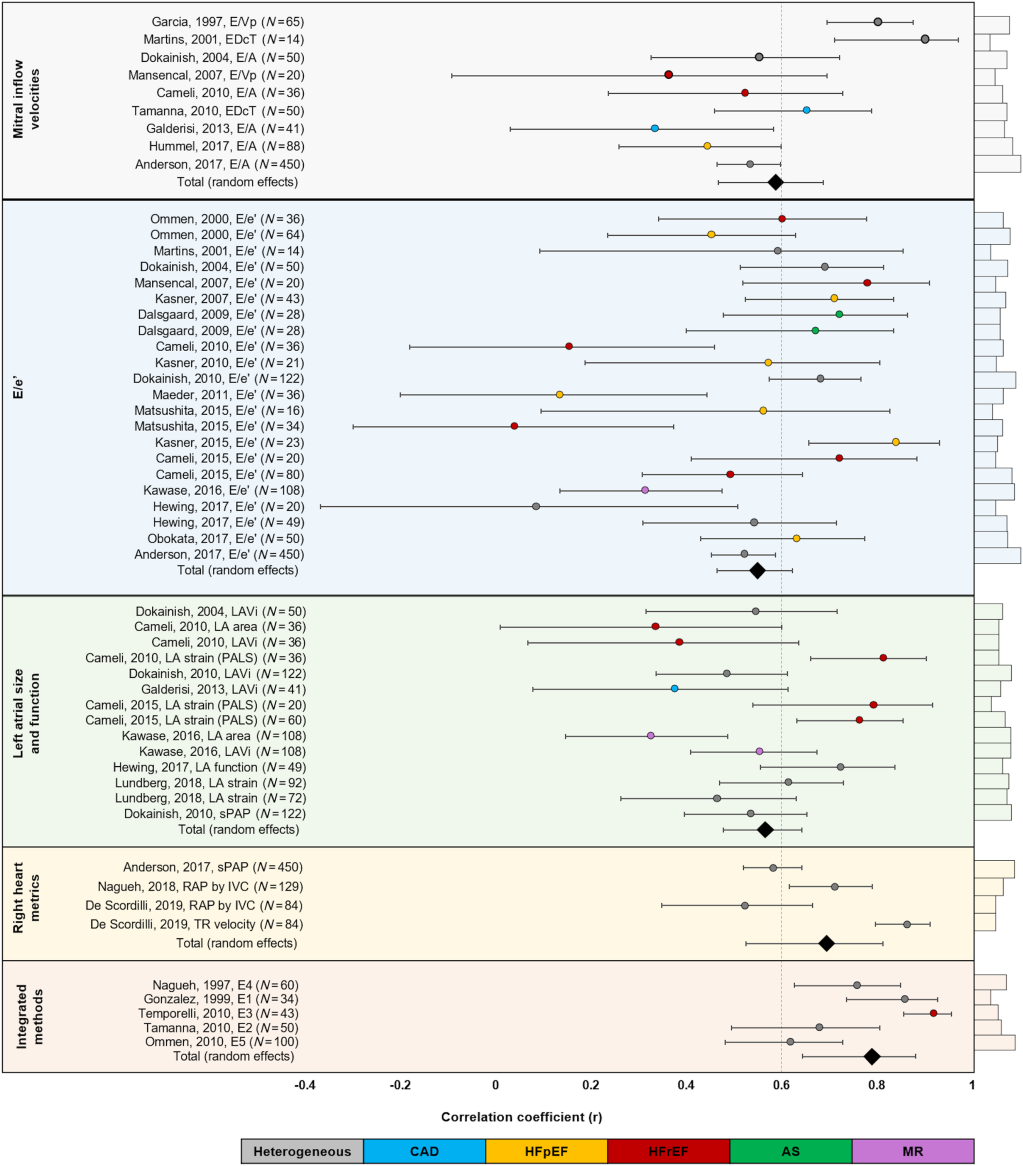
Forest plots of all the methods applied by echocardiography for non‐invasive assessment of left ventricular filling pressure.

The first group, MIVs, had a pooled (random effects) correlation coefficient of 0.58 (95% CI 0.46–0.68, *P* < 0.01) (*Figure* *4*, Supporting Information, *Table* *S5*). Four MIV studies comprised a heterogeneous patient population: two studies investigated HFrEF (*n* = 91), and two investigated coronary artery disease (CAD) (*n* = 56). The study with the highest correlation was that by Martins *et al*. (*r* = 0.90, 95% CI 0.71–0.97).^11^ However, this study had the lowest weighting within this category, recruiting only 14 patients. The most highly weighted study was that by Anderson *et al*. (*n* = 450), showing moderate association between E/A and invasive LVFP (*r* = 0.53).^32^ The most widely studied MIV parameter was E/A (*n* = 665). Other echocardiography metrics studied included E/Vp (*n* = 85) and EDcT (*n* = 64).

The second category, E/e′, was the largest, incorporating 17 studies and 1348 patients [HFpEF, *n* = 253; HFrEF, *n* = 126; heterogeneous, *n* = 785; aortic stenosis (AS), *n* = 28; mitral regurgitation (MR), *n* = 108]. E/e′ had a pooled random effects correlation coefficient of 0.55 (95% CI 0.46–0.62, *P* < 0.01, *z* = 11). Correlation coefficients varied greatly between studies, ranging from 0.08 (95% CI −0.38 to 0.5) (Hewing *et al*.) to 0.84 (95% CI 0.65–0.93) (Kasner *et al*.).^22^, ^24^ The Kawase *et al*. study investigating E/e′ in MR patients (*r* = 0.33) recruited 108 patients, contributing significant weight in reducing the pooled correlation coefficient.^23^

The third major parameter studied was LA size and function. Overall, these studies had a pooled random effects correlation of 0.56 (95% CI 0.47–0.64, *P* < 0.01, *z* = 10, *n* = 952). Of these, LAVi was investigated in five studies, including 357 patients; LA area was also investigated in 144 patients, and LA strain assessments were undertaken in 280 patients. One study evaluated LAVi in MR cases (*n* = 108) and found a correlation coefficient of 0.55 (95% CI 0.40–0.67) (Kawase *et al*.), much stronger than that found for LA area alone (*r* = 0.33, 95% CI 0.00–0.59).^23^ Of the LA parameters, LA strain assessment had the strong correlations, ranging from 0.46 (Lundberg *et al*.) to 0.81 (Cameli *et al*.).

Assessment of the right heart comprised three studies and is the most novel category, with all studies published since 2017. These studies combined had a strong correlation coefficient of 0.69 (95% CI 0.52–0.811 *P* < 0.01, *z* = 6.1, *n* = 747). The largest study was by Anderson *et al*., investigating systolic pulmonary artery pressure (*r* = 0.58, 95% CI 0.52–0.64).^32^ Nagueh *et al*.^27^ and de Scordilli *et al*. investigated right atrial pressure, with the latter also studying TRV.^28^ TRV had a stronger correlation (*r* = 0.86, 95% CI 0.79–0.91), compared with right atrial pressure; however, this is weighted lower due to fewer patient numbers. All studies recruited heterogeneous populations. The five integrated approach studies (*n* = 287) combined several echocardiographic parameters into equations (*Table* *1*) to estimate LVFP. This category had the highest weighted average (random effects) correlation coefficient at 0.79 (95% CI 0.64–0.88, *P* < 0.001, *z* = 6.84). Ommen *et al*. had the highest weighting at 28.6% (*n* = 100) but also had the lowest correlation of all the equations (*r* = 0.62, 95% CI 0.48–0.73) and the lowest clinical applicability of all studies.^10^ In comparison, Temporelli *et al*. found a correlation of 0.92 (95% CI 0.86–0.96) using an equation focusing on MIV and had excellent clinical applicability.^29^ The integrated approach studies incorporated a range of patient cohorts; Nagueh *et al*. and Gonzalez‐Vilchez *et al*. studied heterogeneous populations (*n* = 95), Ommen *et al*. and Temporelli *et al*. recruited HFrEF patients (*n* = 63), Ommen *et al*. further studied 64 patients with HFpEF, and Tamanna *et al*. investigated CAD (*n* = 50).^8^, ^9^, ^10^, ^17^, ^29^

**Table 1:** Integrated equations identified in the literature for estimating left ventricular filling pressure

Abbreviation	First author	Year	*n*	Approach	Disease state
E1	Gonzalez‐Vilchez	1999	54	10^3^/([2·IVRT] + FPV)	Heterogeneous
E2	Tamanna	2010	50	1.43 × DR + 1.32 × E/A − 0.024 × DT + 0.02 × MLAV + 9.2	CAD
E3	Temporelli	2010	43	32.16 + (−0.1045E) + (0.1345A) + (−0.17 DT) + (4.95 E/A)	HFrEF
E4	Nagueh	1997	125	1.91 + (1.24*E/e′)	Heterogeneous
E5	Ommen	2000	100	11.96 + (0.596*E/e′)	HFpEF + HFrEF

CAD, coronary artery disease; DR, deceleration rate; DT, deceleration time; FPV, flow propagation velocity; HFpEF, heart failure with preserved ejection fraction; HFrEF, heart failure with reduced ejection fraction; IVRT, isovolumetric relaxation time; MLAV, maximal left atrial volume.

### Meta‐analysis results for disease

From the 27 eligible studies, five primary patient cohorts were recruited: HFpEF, HFrEF, CAD, AS, and MR. Furthermore, 11 studies did not specify diagnoses, grouped into ‘heterogeneous’ for the purpose of this meta‐analysis.

**Figure 5:**
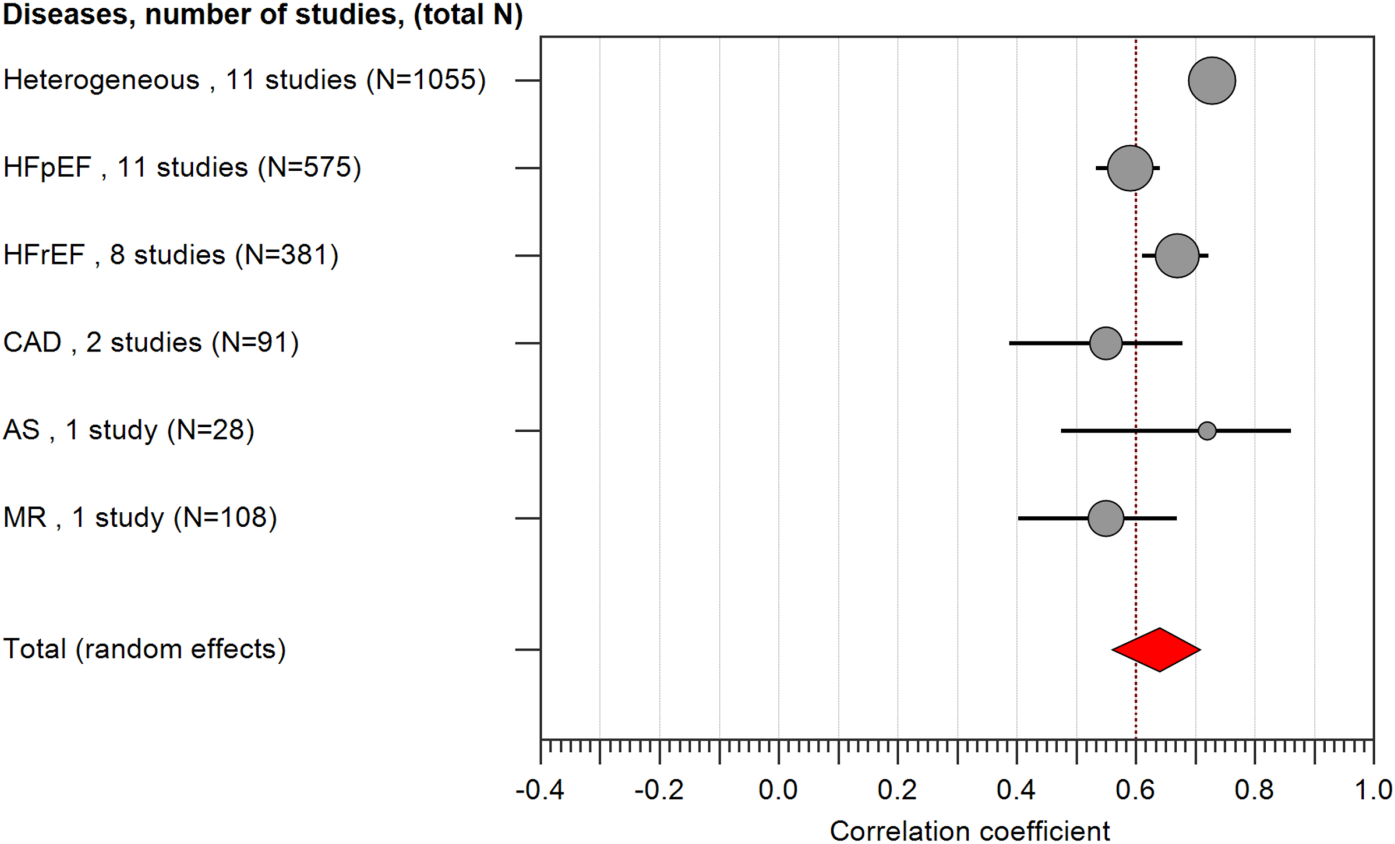
Forest plots of all the heart disease states in which echocardiography has been tested for non‐invasive estimation of left ventricular filling pressure. It is worth noting that the heterogeneous group represented the most patients in the total meta‐analysis (40.6%).

The pooled analysis across all cohorts revealed a modest association between echocardiographic and invasive haemodynamic assessment (*r* = 0.64, 95% CI 0.56–0.71, *P* < 0.01, *z* = 11.9) (*Figure* *5*, Supporting Information, *Table* *S6*).

In total, 1055 patients were categorized as ‘heterogeneous’, and this group demonstrated the best association with the least effect size (*r* = 0.73, 95% CI 0.70–0.76) and highest weighting (22.16%). A small proportion of these patients were intubated in intensive care (*n* = 160). HFpEF (*r* = 0.59, 95% CI 0.53–0.64, *n* = 575) patients were found to have a lower correlation coefficient than HFrEF patients (*r* = 0.67, 95% CI 0.61–0.72, *n* = 381). Patients recruited with a diagnosis of CAD and MR demonstrated the lowest correlation coefficient (*r* = 0.55, 95% CI 0.38–0.68, *n* = 91 and *r* = 0.55, 95% CI 0.40–0.67, *n* = 108, respectively).

### Clinical applicability (Critical Appraisal Skills Programme) results

The modified CASP tool showed that 19% (*n* = 5) of studies were ‘highly clinically applicable’, 56% (*n* = 15) ‘moderately clinically applicable’, and 26% (*n* = 7) ‘less clinically applicable’. Of those ‘less clinically applicable’, most failed to report bias or undertake suitable reproducibility assessments (Supporting Information, *Table* *S2*). Three studies scored 100%; all evaluated a heterogeneous cohort.

## Discussion

This systematic review and meta‐analysis found that non‐invasive echocardiographic estimates of LVFP demonstrate moderate association to invasive right heart catheterization. Five echocardiographic methods were evaluated, highlighting the integrated approach as best correlated. However, evidence for the integrated approach lacked in all HF phenotypes, particularly HFpEF. Of the single approaches, LA strain showed promising results.

### Principal findings

This meta‐analysis demonstrates that echocardiographic approaches to estimating LVFP are feasible and can be applied clinically. However, there was significant heterogeneity in both the quality of studies and their findings. Many studies recruited a heterogeneous cohort of patients, which makes it challenging to apply their methods to predict LVFP in groups of HF patients.

In patients with MR, Kawase *et al*. demonstrated that LA volume and function (minimum LAVi and KT index, a marker of LV function) appears to have a better correlation to invasive PCWP than E/e′.^23^ The main limitation was the poor reproducibility for the novel KT index. In patients with AS, a single small study demonstrated promising results for E/e′ (*r* = 0.72, 95% CI 0.47–0.86), but more evidence is needed to predict LVFP in this cohort. We did not identify any study that has developed personalized non‐invasive models in either mitral stenosis or aortic regurgitation.

Patients with HFpEF represent the second largest group where LVFP has been studied non‐invasively. The pooled correlation with invasive LVFP was modest. Maeder *et al*. demonstrated that E/e′ has a weak association to LVFP in patients with HFpEF.^19^ Two other studies by Hummel *et al*. and by Ommen *et al*. again confirmed a poor correlation of LVEDP to E/e′.^10^, ^25^ The only study showing promising correlation and high clinical applicability within HFpEF was Obokata *et al*. (*r* = 0.63, 95% CI 0.43–0.77, *n* = 50).^26^ They also identified the incremental role of non‐invasive stress E/e′ to improve diagnostic sensitivity.

In HFrEF, the pooled correlation coefficient was much stronger for clinical translation across five studies. However, only one study demonstrated excellent clinical applicability for estimating LVFP by an integrated equation focusing on MIV profile (E3) (Temporelli *et al*.).^29^ The main limitation of this proposed equation is that no validation via a multicentre trial has been undertaken to support widespread clinical adoption. Cameli *et al*. demonstrated a strong correlation using LA strain parameters in HFrEF in a small study of 36 patients. In patients with CAD, the pooled association was only modest, and both studies that specifically recruited CAD patients demonstrated only medium to limited clinical applicability.

Further, our study is unique due to its prominent inclusion of heterogeneous patient populations, consisting of a number of cardiac pathologies as well as patients with normal LVFP. While the algorithm laid out in ASE/EACVI 2016 recommendations excludes patients without cardiac pathologies,^6^ we decided a heterogeneous cohort better matches that seen in clinical practice, where diagnoses are often unclear at the time the algorithm is applied.^34^ This decision is supported by our finding that the most clinically applicable methods comprised the heterogeneous cohorts.^7^, ^8^, ^9^, ^28^ These studies demonstrated good association and scored high on the modified CASP. However, one of these studies was on only intubated patients.^7^ Nonetheless, Nagueh *et al*. recruited a combination of intubated and non‐intubated patients to derive their non‐invasive equation and overall, demonstrated a high correlation coefficient of 0.87.^8^ However, overall, there is a lack of clarity of evidence in HFpEF patients supporting the routine clinical use of E/e′ as an accurate estimate of LVFP.

### Comparisons with other recent meta‐analyses

Sharifov *et al*. undertook a systematic review and meta‐analysis of 24 studies investigating the correlation between invasive LVFP using non‐invasive E/e′ in patients with HFpEF.^35^ However, there are differences between ours and the Sharifov *et al*. work. Firstly, the majority of their included studies did not perform simultaneous echocardiography and right heart catheterization. Our study has addressed this issue, with 26/27 of our included studies having simultaneous investigation. Sharifov *et al*. concluded a lack of evidence for a substantive correlation with E/e′, requiring further validation, and this statement is supported by our findings. Furthermore, Nauta *et al*. also performed a systematic review studying the correlation between echocardiographically estimated and invasively measured LVFP in patients with HFpEF and again found a modest correlation using the E/e′ method.^36^ This study further considered the prognostic relevance of this, demonstrating improvements when using multiple parameters simultaneously. These conclusions strengthen our findings on the utility of integrated approaches. These studies, however, are limited to HFpEF cohorts. Our meta‐analysis has divided patients by a variety of diagnoses, providing a broader view of the usefulness of echocardiography in estimating LVFP in HF.

### Context, implications for health policy, and future research

It is clear that one single non‐invasive echocardiographic parameter cannot reliably estimate LVFP across all cardiovascular pathologies. Importantly, in HFpEF, where it is critically important to have such a non‐invasive diagnostic tool, we have very limited evidence to support any current single or even integrated method by echocardiography. This meta‐analysis supports previous publications calling for further research to validate non‐invasive LVFP estimates in HFpEF. Future studies should consider robust clinical validation including reproducibility tests, which was identified as one of the reasons why some studies were less clinically applicable. When reporting correlation coefficients, the studies should also report either bias or standard error, between non‐invasive and invasive methods. In patients with HFrEF, the evidence is much stronger, and a cautious approach to inform LVFP can be applied.

### Limitations

In this systematic review and meta‐analysis, we did not include studies specifically looking at the diagnostic accuracy of any echocardiographic metric or integrated equation on a categorical scale. Hence, we did not report studies that have performed sensitivity/specificity analysis. We did this intentionally to investigate the true continuum of any non‐invasive method vs. the reference invasive method. In addition, this work did not evaluate the prognostic role of non‐invasive echocardiographic parameters.

## Conclusions

Pooled echocardiographic indices show moderate association to invasively measured LVFP; however, this varies with cardiovascular pathology. In HFpEF or valvular heart disease, no single echocardiography measure offers a reliable estimate of LVFP. In HFrEF patients, mitral inflow‐derived indices demonstrate promise for routine clinical use. Most studies included heterogeneous patient groups and thus cannot be applied to a precision medicine approach. Integrated methods incorporating several echocardiographic metrics appear most promising for estimating LVFP reliably but require further validation in larger, patient‐specific studies.

## Conflict of interest

None declared.

## Funding

This work was supported by Wellcome Trust grants (A.R.—206632/Z/17/Z), (P.G.‐220703/Z/20/Z) and (A.J.S.—205188/Z/16/Z). L.Z. was supported by the National Medical Research Council (NMRC/OFIRG/0018/2016). P.G. was supported by the Academy of Sciences Starter Grant (SGL018\1100).

## Supporting information


**Table S1.** The modified CASP questions used in the systematic review process.
**Table S2.** Full results of the Modified Critical Appraisal Skills Programme (CASP) tool results.
**Table S3.** Studies reporting bias between invasively measured versus non‐invasively predicted LVEDP/PCWP.
**Table S4.** All the 27 studies which were pooled together in systematic review for further meta‐analysis with respective random effects weighting in percentage (%).
**Table S5**. Data of all echocardiographic methods studied for their association to LV filling pressure.
**Table S6.** Supplementary data to Figure 3a showing correlation between echocardiographically estimated LVEDP and invasive measurements in different disease states.

## Data Availability

All data relevant to the study are included in the article or uploaded as supporting information.
