# Supplementary material for: Meta‐analysis of echocardiographic quantification of left ventricular filling pressure
Source: ESC Heart Fail. 2020 Nov 23;8(1):566–76. doi: 10.1002/ehf2.13119 (PMC7835555; doi:10.1002/ehf2.13119)
Supplement: Supplementary file 1 — Table S1. The modified CASP questions used in the systematic review process. Table S2. Full results of the Modified Critical Appraisal Skills Programme (CASP) tool results. Table S3. Studies reporting bias between invasively measured versus non‐invasively predicted LVEDP/PCWP. Table S4. All the 27 studies which were pooled together in systematic review for further meta‐analysis with respective random effects weighting in percentage (%). Table S5. Data of all echocardiographic methods studied for their association to LV filling pressure. Table S6. Supplementary data to Figure 3a showing correlation between echocardiographically estimated LVEDP and invasive measurements in different disease states. [file EHF2-8-566-s001.docx]

**Supplementary Online Document**

Index

1. Abbreviations
2. Detailed Search Strategy
3. Statistical Methods
4. Modified CASP questions for testing clinical applicability
5. Supplementary table 1
6. Supplementary table 2
7. Supplementary table 3
8. Supplementary table 4
9. Supplementary table 5
10. Supplementary table 6

**Abbreviations**

CAD Coronary Artery Disease

CI Confidence Interval

EDcT E wave Deceleration Time

HF Heart Failure

HFpEF Heart Failure with preserved Ejection Fraction

HFrEF Heart Failure with reduced Ejection Fraction

LAVi Left Atrial Volume indexed

LVEDP Left Ventricular End Diastolic Pressure

LVFP Left Ventricular Filling Pressure

MIV Mitral Inflow Velocities

MR Mitral Regurgitation

PALS Peak Atrial Longitudinal Strain

PCWP Pulmonary Capillary Wedge Pressure

PRISMA Preferred Reporting Items for Systematic Reviews and Meta-Analyses

RAP Right Atrial Pressure

TRV Tricuspid Regurgitation Velocity

TTE Transthoracic Echocardiography

VHD Valvular Heart Disease

Vp Propagation Velocity

**Detailed Search Strategy**

The following search was undertaken on the Scopus database on 21/11/2019:

( TITLE-ABS-KEY ( invasive ) AND TITLE-ABS-KEY (noninvasive OR echocardiography ) AND TITLE-ABS-KEY ( lvedp OR pulmonary AND capillary AND wedge AND pressure OR pcwp ) ) AND DOCTYPE ( ar ) AND ( LIMIT-TO ( DOCTYPE , "ar" ) ) AND ( LIMIT-TO ( SUBJAREA , "MEDI" ) ) AND ( LIMIT-TO ( LANGUAGE , "English" )

The Scopus database includes over 22,800 publications including over 21,950 peer reviewed journals and 'articles in press' from over 8000 journals. This search returned 197 results. The results were screened and the following inclusion criteria applied: *'Medicine’* category; in English; on humans ≥18 years; in the final publication stage, >12 patients. Two independent assessors reviewed the 207 subsequent papers and 23 were agreed to be included in our study.

Further, a comprehensive review of all the referenced literature was undertaken and identified an additional 11 results. Duplicates (n=1) were removed, leaving 10 studies. The above exclusion criteria was applied and all full text articles reviewed by both assessors. In total, 27 research outcome papers were included in this study.

**Statistical Methods**

For each study, if reported, we also pooled in the disease-specific r-values and numbers recruited in that disease state. The Hedges-Olkin method was used to calculate the weighted summary correlation coefficient under the fixed effects model, using a Fisher Z transformation of the correlation coefficients. Next, the heterogeneity statistics were incorporated to calculate the summary correlation coefficient under the random-effects model with 95% confidence interval (CI) was used in all analyses. The main findings of the meta-analysis were presented as Forest plots and tables. We used the I² statistic to measure heterogeneity among the studies in each analysis and followed the recommendations for thresholds outlined in Section 9.5.2 of the Cochrane Handbook for Systematic Reviews of Interventions.

**Modified CASP questions for testing clinical applicability**

The modified CASP questionnaire included eight questions; answers of ‘yes’ scored one point whilst answers of ‘no’ or ‘unknown’ scored zero points. Section A of the tool remained unchanged and formed questions 1 to 6 in our modified tool. Section B was combined into a single question assessing bias and formed question 7 in our tool. Section C was omitted. In addition to these changes, we added two questions (questions 8 and 9) in order to further assess reproducibility and impact of the included studies. Reproducibility has been adjusted for historic validation by previous studies. For example, after the reproducibility was established by the Mansencal *et al* study for E/e’, future studies didn’t need to repeat this and automatically got a score of 1. The automatic score happened only from next year.

In addition to questions, we used a component of meta-analysis to improve the precision of the clinical applicability. This was done by deriving the random effect weighting (%) of all studies finally included in the meta-analysis. The average weighting was used as a threshold of 3.7%. Any study with a weighting of ≥3.7% and had a minimum correlation coefficient of 0.6 to invasive LVFP scored one additional point.

Ultimately, we used 9 questions for our modified CASP tool. Each question carried a score of 1. The total score out of 9 was converted to a percentage, the clinical applicability score. These scores were split into three categories: ‘highly clinically applicable’ for a score of 80-100%, ‘moderately clinically applicable’ for 70-79%, and ‘less clinically applicable’ for <69%.

**Supplementary Table 1.** The modified CASP questions used in the systematic review process.

| Q1: Was there a clear question for the study to address? |
| --- |
| Q2: Was there a comparison with an appropriate reference standard? |
| Q3: Did all participants receive both the diagnostic and the reference test? |
| Q4: Are the results of the test free from influence from the results of the reference standard? |
| Q5: Is the disease state of the tested population clearly described? |
| Q6: Were the methods for performing the test described in sufficient detail? |
| Q7: Has the study reported bias? |
| Q8: Did the study meet or exceed the weight threshold (4.3%) and did it demonstrate correlation coefficient of equal to or greater than 0.6? |
| Q9: Was a reproducibility assessment performed? |

**Supplementary Table 2.** Full results of the Modified Critical Appraisal Skills Programme (CASP) tool results.

| **Author** | **Year** | **Metric** | **Q1** | **Q2** | **Q3** | **Q4** | **Q5** | **Q6** | **Q7** | **Q8** | **Q9** | **CA (%)** |
| --- | --- | --- | --- | --- | --- | --- | --- | --- | --- | --- | --- | --- |
| Garcia | 1997 | E/Vp | 1 | 1 | 1 | 1 | 1 | 1 | 1 | 1 | 1 | 100 |
| Nagueh | 1997 | E4 | 1 | 1 | 1 | 1 | 1 | 1 | 1 | 1 | 1 | 100 |
| De Scordilli | 2019 | TR velocity | 1 | 1 | 1 | 1 | 1 | 1 | 1 | 1 | 1 | 100 |
| Temporelli | 2010 | E3 | 1 | 1 | 1 | 1 | 1 | 1 | 1 | 0 | 1 | 89 |
| Obokata | 2017 | E/e' | 1 | 1 | 1 | 1 | 1 | 1 | 0 | 1 | 1 | 89 |
| Gonzalez | 1999 | E1 | 1 | 1 | 1 | 1 | 1 | 1 | 0 | 0 | 1 | 78 |
| Martins | 2001 | EDcT | 1 | 1 | 1 | 1 | 1 | 1 | 1 | 0 | 0 | 78 |
| Dokainish | 2004 | E/e' | 1 | 1 | 1 | 1 | 1 | 1 | 0 | 1 | 0 | 78 |
| Mansencal | 2007 | E/e' | 1 | 1 | 1 | 1 | 1 | 1 | 0 | 0 | 1 | 78 |
| Cameli | 2010 | LA function | 1 | 1 | 1 | 0 | 1 | 1 | 1 | 0 | 1 | 78 |
| Kasner | 2010 | E/e' | 1 | 1 | 1 | 1 | 1 | 1 | 0 | 0 | 1 | 78 |
| Dokainish | 2010 | E/e' | 1 | 1 | 1 | 1 | 1 | 1 | 0 | 1 | 0 | 78 |
| Maeder | 2011 | E/e' | 1 | 1 | 1 | 1 | 1 | 1 | 1 | 0 | 0 | 78 |
| Kasner | 2015 | E/e' | 1 | 1 | 1 | 1 | 1 | 1 | 0 | 0 | 1 | 78 |
| Cameli | 2015 | LA strain | 1 | 1 | 1 | 0 | 1 | 1 | 0 | 1 | 1 | 78 |
| Kawase | 2016 | LAVi | 1 | 1 | 1 | 1 | 1 | 1 | 1 | 0 | 0 | 78 |
| Hewing | 2017 | LA function | 1 | 1 | 1 | 1 | 1 | 1 | 0 | 1 | 0 | 78 |
| Nagueh | 2018 | RAP by IVC | 1 | 1 | 1 | 1 | 0 | 1 | 0 | 1 | 1 | 78 |
| Lundberg | 2018 | LA strain | 1 | 1 | 1 | 0 | 1 | 1 | 0 | 1 | 1 | 78 |
| Anderson | 2018 | sPAP | 1 | 1 | 1 | 1 | 1 | 1 | 0 | 0 | 1 | 78 |
| Kasner | 2007 | E/e' | 1 | 1 | 1 | 1 | 1 | 1 | 0 | 0 | 0 | 67 |
| Tamanna | 2010 | E2 | 1 | 1 | 1 | 0 | 1 | 1 | 0 | 1 | 0 | 67 |
| Galderisi | 2013 | LAVi | 1 | 1 | 1 | 1 | 1 | 1 | 0 | 0 | 0 | 67 |
| Hummel | 2017 | E/A | 1 | 1 | 1 | 1 | 1 | 1 | 0 | 0 | 0 | 67 |
| Dalsgaard | 2009 | E/e' | 1 | 1 | 1 | 1 | 0 | 1 | 0 | 0 | 0 | 56 |
| Matsushita | 2015 | E/e' | 1 | 1 | 1 | 0 | 1 | 1 | 0 | 0 | 0 | 56 |
| Ommen | 2000 | E5 | 1 | 1 | 1 | 0 | 0 | 1 | 0 | 0 | 0 | 44 |

**Abbreviations:** Q1-Q9=Question 1 to Question 9 - for complete detail on each question refer to supplementary Table 1. CA, clinical applicability; Vp, propagation velocity; E4, equation 4; E3, equation 3; TR, tricuspid regurgitation; E1, equation 1; EDcT, deceleration time of E; LA, left atrial; LAVi, left atrial volume indexed; RAP, right atrial pressure; IVC, inferior vena cava; sPAP, systolic pulmonary artery pressure; E2, equation 2; E5, equation 5.

**Supplementary Table 3.** Studies reporting bias between invasively measured versus non-invasively predicted LVEDP/PCWP.

| **First Author** | **Year** | **N** | **Disease State** | **Reported Bias** |
| --- | --- | --- | --- | --- |
| Garcia | 1997 | 65 | Heterogeneous | 1±3 mmHg |
| Nagueh | 1997 | 125 | Heterogeneous | 0.1±4 mmHg |
| Martins | 2001 | 14 | Heterogeneous | 0.4±7mmHg |
| Cameli | 2010 | 36 | HFrEF | 0.1±8mmHg |
| Temporelli | 2010 | 43 | HFrEF | 0.2±6mmHg |
| Maeder | 2011 | 36 | HFpEF | 9±9mmHg |
| Kawase | 2016 | 108 | MR | 2±5mmHg |
| De Scordilli | 2019 | 84 | Heterogeneous | -2±13mmHg |

**Abbreviations:** HFrEF, heart failure with reduced ejection fraction; HFpEF, heart failure with preserved ejection fraction; MR, mitral regurgitation.

**Supplementary Table 4.** All the 27 studies which were pooled together in systematic review for further meta-analysis with respective random effects weighting in percentage (%).

| **Study** | **N** | **R** | **95% CI** | **z** | **P** | **Weight (%)** |
| --- | --- | --- | --- | --- | --- | --- |
| Garcia, 1997, E/Vp, (N=65) | 65 | 0.8 | 0.691 to 0.873 |  |  | 3.97 |
| Nagueh, 1997, E4, (N=60) | 60 | 0.76 | 0.627 to 0.850 |  |  | 3.9 |
| Gonzalez, 1999, E1, (N=34) | 34 | 0.86 | 0.736 to 0.928 |  |  | 3.3 |
| Ommen, 2000, E5, (N=100) | 100 | 0.53 | 0.372 to 0.658 |  |  | 4.29 |
| Martins, 2001, EDcT, (N=14) | 14 | 0.9 | 0.707 to 0.968 |  |  | 2.05 |
| Dokainish, 2004, E/e', (N=50) | 50 | 0.69 | 0.510 to 0.812 |  |  | 3.73 |
| Mansencal, 2007, E/e', (N=20) | 20 | 0.78 | 0.515 to 0.909 |  |  | 2.59 |
| Kasner, 2007, E/e', (N=43) | 43 | 0.71 | 0.521 to 0.833 |  |  | 3.57 |
| Dalsgaard, 2009, E/e', (N=28) | 28 | 0.72 | 0.474 to 0.862 |  |  | 3.06 |
| Cameli, 2010, LA function (PALS), (N=36) | 36 | 0.81 | 0.656 to 0.899 |  |  | 3.37 |
| Tamanna, 2010, E2, (N=50) | 50 | 0.68 | 0.495 to 0.806 |  |  | 3.73 |
| Temporelli, 2010, E3, (N=43) | 43 | 0.92 | 0.856 to 0.956 |  |  | 3.57 |
| Kasner, 2010, E/e', (N=21) | 21 | 0.57 | 0.183 to 0.804 |  |  | 2.66 |
| Dokainish, 2010, E/e', (N=122) | 122 | 0.68 | 0.571 to 0.765 |  |  | 4.4 |
| Maeder, 2011, E/e', (N=36) | 36 | 0.13 | -0.207 to 0.440 |  |  | 3.37 |
| Galderisi, 2013, LAVi, (N=41) | 41 | 0.37 | 0.07 to 0.60 |  |  | 3.52 |
| Matsushita, 2015, E/e', (N=50) | 50 | 0.26 | -0.019 to 0.5 |  |  | 3.73 |
| Kasner, 2015, E/e', (N=23) | 23 | 0.84 | 0.654 to 0.930 |  |  | 2.79 |
| Cameli, 2015, LA strain, (N=80) | 80 | 0.77 | 0.662 to 0.847 |  |  | 4.14 |
| Kawase, 2016, LAVi, (N=108) | 108 | 0.55 | 0.403 to 0.669 |  |  | 4.33 |
| Hewing, 2017, LA function, (N=69) | 69 | 0.72 | 0.583 to 0.817 |  |  | 4.02 |
| Hummel, 2017, E/A, (N=88) | 88 | 0.44 | 0.254 to 0.595 |  |  | 4.2 |
| Obokata, 2017, E/e', (N=50) | 50 | 0.63 | 0.426 to 0.773 |  |  | 3.73 |
| Nagueh, 2018, RAP by IVC, (N=129) | 129 | 0.71 | 0.612 to 0.786 |  |  | 4.43 |
| Lundberg, 2018, LA strain, (N=164) | 164 | 0.61 | 0.504 to 0.698 |  |  | 4.54 |
| Anderson, 2018, sPAP, (N=450) | 450 | 0.58 | 0.515 to 0.638 |  |  | 4.81 |
| De Scordilli, 2019, TR velocity, (N=84) | 84 | 0.86 | 0.792 to 0.907 |  |  | 4.17 |
| Total (fixed effects) | 2058 | 0.66 | 0.634 to 0.684 | 35.2 | <0.01 | 100 |
| Total (random effects) | 2058 | 0.69 | 0.629 to 0.745 | 15.1 | <0.01 | 100 |
| **Test for heterogeneity** |  |  |  |  |  |  |
| Q | 140.98 |  |  |  |  |  |
| DF | 26 |  |  |  |  |  |
| Significance level | P<0.01 |  |  |  |  |  |
| I2 (inconsistency) | 81.6% |  | 95% CI | 74 | to | 86.9 |

**Supplementary Table 5**. Data of all echocardiographic methods studied for their association to LV filling pressure.

| **Study** | **N** | **R** | **95% CI** | **z** | **P** | **W (%)** |
| --- | --- | --- | --- | --- | --- | --- |
| Mitral inflow Doppler based methods | | | | | | |
| Garcia, 1997, E/Vp (N=65) | 65 | 0.8 | 0.691 to 0.873 |  |  | 12.7 |
| Martins, 2001, EDcT (N=14) | 14 | 0.9 | 0.707 to 0.968 |  |  | 5.6 |
| Dokainish, 2004, E/A (N=50) | 50 | 0.55 | 0.321 to 0.718 |  |  | 11.6 |
| Mansencal, 2007, E/Vp (N=20) | 20 | 0.36 | -0.098 to 0.69 |  |  | 7.3 |
| Cameli, 2010, E/A (N=36) | 36 | 0.52 | 0.231 to 0.725 |  |  | 10.2 |
| Tamanna, 2010, EDcT (N=50) | 50 | 0.65 | 0.454 to 0.786 |  |  | 11.6 |
| Galderisi, 2013, E/A (N=41) | 41 | 0.33 | 0.025 to 0.58 |  |  | 10.8 |
| Hummel, 2017, E/A (N=88) | 88 | 0.44 | 0.254 to 0.595 |  |  | 13.7 |
| Anderson, 2017, E/A (N=450) | 450 | 0.53 | 0.460 to 0.593 |  |  | 16.6 |
| Total (random effects) | 814 | 0.584 | 0.462 to 0.684 | 7.8 | <0.01 | 100.0 |
| Test for heterogeneity |  |  |  |  |  |  |
| Q | 29.54 | DF | 8 |  |  |  |
| Significance level | P = 0.03 |  |  |  |  |  |
| I2 (inconsistency) | 72.92% | 95% CI | 47.01 to 86.16 |  |  |  |
| E/e' | | | | | | |
| Ommen, 2000, E/e' (N=36) | 36 | 0.6 | 0.338 to 0.776 |  |  | 4.5 |
| Ommen, 2000, E/e' (N=64) | 64 | 0.45 | 0.230 to 0.627 |  |  | 5.6 |
| Martins, 2001, E/e' (N=14) | 14 | 0.59 | 0.086 to 0.85 |  |  | 2.5 |
| Dokainish, 2004, E/e' (N=50) | 50 | 0.69 | 0.510 to 0.812 |  |  | 5.2 |
| Mansencal, 2007, E/e' (N=20) | 20 | 0.78 | 0.515 to 0.909 |  |  | 3.3 |
| Kasner, 2007, E/e' (N=43) | 43 | 0.71 | 0.521 to 0.833 |  |  | 4.9 |
| Dalsgaard, 2009, E/e' (N=28) | 28 | 0.72 | 0.474 to 0.862 |  |  | 4.0 |
| Dalsgaard, 2009, E/e' (N=28) | 28 | 0.67 | 0.396 to 0.834 |  |  | 4.0 |
| Cameli, 2010, E/e' (N=36) | 36 | 0.15 | -0.188 to 0.456 |  |  | 4.5 |
| Kasner, 2010, E/e' (N=21) | 21 | 0.57 | 0.183 to 0.804 |  |  | 3.4 |
| Dokainish, 2010, E/e' (N=122) | 122 | 0.68 | 0.571 to 0.765 |  |  | 6.5 |
| Maeder, 2011, E/e' (N=36) | 36 | 0.13 | -0.207 to 0.440 |  |  | 4.5 |
| Matsushita, 2015, E/e' (N=16) | 16 | 0.56 | 0.089 to 0.826 |  |  | 2.8 |
| Matsushita, 2015, E/e' (N=34) | 34 | 0.035 | -0.307 to 0.369 |  |  | 4.4 |
| Kasner, 2015, E/e' (N=23) | 23 | 0.84 | 0.654 to 0.930 |  |  | 3.6 |
| Cameli, 2015, E/e' (N=20) | 20 | 0.72 | 0.407 to 0.882 |  |  | 3.3 |
| Cameli, 2015, E/e' (N=80) | 80 | 0.49 | 0.303 to 0.641 |  |  | 5.9 |
| Kawase, 2016, E/e' (N=108) | 108 | 0.31 | 0.129 to 0.471 |  |  | 6.3 |
| Hewing, 2017, E/e' (N=20) | 20 | 0.08 | -0.376 to 0.505 |  |  | 3.3 |
| Hewing, 2017, E/e' (N=49) | 49 | 0.54 | 0.305 to 0.713 |  |  | 5.1 |
| Obokata, 2017, E/e' (N=50) | 50 | 0.63 | 0.426 to 0.773 |  |  | 5.2 |
| Anderson, 2017, E/e' (N=450) | 450 | 0.52 | 0.449 to 0.584 |  |  | 7.3 |
| Total (random effects) | 1348 | 0.546 | 0.461 to 0.620 | 11 | <0.01 | 100.0 |
| Test for heterogeneity |  |  |  |  |  |  |
| Q | 67.89 | DF | 21 |  |  |  |
| Significance level | P<0.01 |  |  |  |  |  |
| I2 (inconsistency) | 69.07% | 95% CI | 52.06 to 80.04 |  |  |  |
| Left atrial size and function | | | | | | |
| Dokainish, 2004, LAVi (N=50) | 50 | 0.54 | 0.308 to 0.711 |  |  | 6.8 |
| Cameli, 2010, LA area (N=36) | 36 | 0.33 | 0.0016 to 0.59 |  |  | 5.9 |
| Cameli, 2010, LAVi (N=36) | 36 | 0.38 | 0.059 to 0.63 |  |  | 5.9 |
| Cameli, 2010, LA strain (N=36) | 36 | 0.81 | 0.656 to 0.899 |  |  | 5.9 |
| Dokainish, 2010, LAVi (N=122) | 122 | 0.48 | 0.330 to 0.606 |  |  | 8.9 |
| Galderisi, 2013, LAVi (N=41) | 41 | 0.37 | 0.070 to 0.61 |  |  | 6.3 |
| Cameli, 2015, LA strain (N=20) | 20 | 0.79 | 0.534 to 0.913 |  |  | 4.1 |
| Cameli, 2015, LA strain(N=60) | 60 | 0.76 | 0.627 to 0.850 |  |  | 7.3 |
| Kawase, 2016, LA area (N=108) | 108 | 0.32 | 0.139 to 0.480 |  |  | 8.7 |
| Kawase, 2016, LAVi (N=108) | 108 | 0.55 | 0.403 to 0.669 |  |  | 8.7 |
| Hewing, 2017, LA function (N=49) | 49 | 0.72 | 0.550 to 0.833 |  |  | 6.8 |
| Lundberg, 2018, LA strain (N=92) | 92 | 0.61 | 0.463 to 0.724 |  |  | 8.3 |
| Lundberg, 2018, LA strain (N=72) | 72 | 0.46 | 0.256 to 0.625 |  |  | 7.8 |
| Dokainish, 2010, sPAP (N=122) | 122 | 0.53 | 0.389 to 0.647 |  |  | 8.9 |
| Total (random effects) | 952 | 0.559 | 0.471 to 0.637 | 10 | <0.01 | 100.0 |
| Test for heterogeneity |  |  |  |  |  |  |
| Q | 41.517 | DF | 13 |  |  |  |
| Significance level | P=0.01 |  |  |  |  |  |
| I2 (inconsistency) | 68.69% | 95% CI | 45.54 to 82.00 |  |  |  |
| Right heart assessment | | | | | | |
| Anderson, 2017, sPAP (N=450) | 450 | 0.58 | 0.515 to 0.638 |  |  | 27.2 |
| Nagueh, 2018, RAP by IVC (N=129) | 129 | 0.71 | 0.612 to 0.786 |  |  | 25.2 |
| De Scordilli, 2019, RAP by IVC (N=84) | 84 | 0.52 | 0.344 to 0.661 |  |  | 23.8 |
| De Scordilli, 2019, TR velocity (N=84) | 84 | 0.86 | 0.792 to 0.907 |  |  | 23.8 |
| Total (random effects) | 747 | 0.69 | 0.520 to 0.808 | 6.1 | <0.01 | 100.0 |
| Test for heterogeneity |  |  |  |  |  |  |
| Q | 32.063 | DF | 3 |  |  |  |
| Significance level | P<0.01 |  |  |  |  |  |
| I2 (inconsistency) | 90.64% | 95% CI | 79.08 to 95.81 |  |  |  |
| Integrated equations | | | | | | |
| Nagueh, 1997, E4 (N=60) | 60 | 0.76 | 0.627 to 0.850 |  |  | 20.6 |
| Gonzalez, 1999, E1 (N=34) | 34 | 0.86 | 0.736 to 0.928 |  |  | 18.3 |
| Temporelli, 2010, E3 (N=43) | 43 | 0.92 | 0.856 to 0.956 |  |  | 19.4 |
| Tamanna, 2010, E2 (N=50) | 50 | 0.68 | 0.495 to 0.806 |  |  | 19.9 |
| Ommen, 2010, E5 (N=100) | 100 | 0.62 | 0.482 to 0.728 |  |  | 21.9 |
| Total (random effects) | 287 | 0.79 | 0.644 to 0.881 | 6.8 | <0.01 | 100.0 |
| Test for heterogeneity |  |  |  |  |  |  |
| Q | 25.25 | DF | 4 |  |  |  |
| Significance level | P<0.01 |  |  |  |  |  |
| I2 (inconsistency) | 84.15% | 95% CI | 64.38 to 92.95 |  |  |  |

**Abbreviations:** U, upper; L, lower; CI, confidence interval; Vp, propagation velocities; EDcT, E wave deceleration time; DF, degrees of freedom; LAVi, left atrial volume indexed; LA, left atrial; PALS, peak atrial longitudinal strain; RAP, right atrial pressure; IVC, inferior vena cava; TRV, tricuspid regurgitation velocity.

^<^Heart failure with reduced ejection fraction, ^>^Heart failure with preserved ejection fraction, *E/e’ (lateral), ^#^Septal E/e’ (septal)

**Supplementary Table 6.** Supplementary data to Figure 3a showing correlation between echocardiographically estimated LVEDP and invasive measurements in different disease states.

| Disease state, number of studies, (total N) | N | R | 95% CI | z | P | Weight (%) |
| --- | --- | --- | --- | --- | --- | --- |
| Heterogeneous , 11 studies (N=1055) | 1055 | 0.73 | 0.698 to 0.755 |  |  | 22.16 |
| HFpEF , 11 studies (N=575) | 575 | 0.59 | 0.534 to 0.641 |  |  | 21.24 |
| HFrEF , 8 studies (N=381) | 381 | 0.67 | 0.611 to 0.722 |  |  | 20.29 |
| CAD , 2 studies (N=91) | 91 | 0.55 | 0.388 to 0.679 |  |  | 14.13 |
| AS , 1 study (N=28) | 28 | 0.72 | 0.474 to 0.862 |  |  | 7.08 |
| MR , 1 study (N=108) | 108 | 0.55 | 0.403 to 0.669 |  |  | 15.1 |
| Total (fixed effects) | 2238 | 0.672 | 0.649 to 0.694 | 38.4 | <0.01 | 100 |
| Total (random effects) | 2238 | 0.64 | 0.560 to 0.708 | 11.9 | <0.01 | 100 |
| Test for heterogeneity |  |  |  |  |  |  |
| Q | 31 |  |  |  |  |  |
| DF | 5 |  |  |  |  |  |
| Significance level | P<0.01 |  |  |  |  |  |
| I2 (inconsistency) | 83.91% | 95% CI | 66.50 to 92.27 |  |  |  |

Abbreviations: CI, confidence interval; HFpEF, heart failure with preserved ejection fraction; HFrEF, heart failure with reduced ejection fraction; CAD, coronary artery disease; AS, aortic stenosis; MR, mitral regurgitation; DF, degrees of freedom
